# Anterior Chest Wall Reconstruction After Total Sternectomy for Solitary Metastasis From Thyroid Carcinoma: A Case Report of a Wire-Only Technique

**DOI:** 10.7759/cureus.109778

**Published:** 2026-05-27

**Authors:** Heemel Saha, Kazi Shameemus Salam, Sanjoy Kumar Saha, SMA Zulker Nine, Karim Hossain

**Affiliations:** 1 Cardiac Surgery (Thoracic Surgery Unit), Bangladesh Medical University, Dhaka, BGD; 2 Otolaryngology - Head, Neck Surgery, Bangladesh Medical University, Dhaka, BGD; 3 Cardiac Anaethesia, Bangladesh Medical University, Dhaka, BGD

**Keywords:** chest wall reconstruction, donati stitches, robicsek wire technique, total sternectomy, wire-only

## Abstract

Solitary metastasis of papillary thyroid carcinoma in the sternum is rare. The management and reconstruction of such cases pose a challenge. Surgical removal with clear margins provides local control and relieves the symptoms. However, chest wall reconstruction after total sternectomy remains a complex procedure. A 40-year-old man presented with a painful anterior chest wall mass and a thyroid nodule. Positron emission tomography-computed tomography (PET-CT) revealed a solitary hypermetabolic lytic lesion involving the sternum and a Fluorodeoxyglucose (FDG) - a radioactive glucose analog used primarily as a radiotracer in PET scan- avid lesion in the right thyroid lobe. Biopsy confirmed the presence of malignant papillary thyroid cancer. After undergoing multidisciplinary evaluation, the patient underwent a six-session chemotherapy regimen of paclitaxel-carboplatin; restaging showed no evidence of extra-metastatic disease. The patient underwent total thyroidectomy, followed by radical ‘en bloc’ total sternectomy with resection of the manubrium, sternal body, xiphoid, and medial ends of both clavicles and first to seventh costal cartilages. Utilising a modified Robicsek stainless steel wire-only technique, a 15×10 cm anterior chest wall defect was reconstructed with a semi-rigid neosternum without any prosthetic mesh or muscle flap. The patient was extubated after 6 h, showed no paradoxical respiration or chest wall instability, and achieved primary healing. One month later, the patient showed complete functional recovery. Total sternectomy with modified Robicsek wire-only reconstruction is a safe, effective, and economical technique in patients with isolated sternal metastases from papillary thyroid cancer (PTC). This method is especially beneficial for patients whose prosthetic materials are prohibitively expensive or unavailable.

## Introduction

Sternal metastasis from papillary thyroid carcinoma is uncommon but aggressive [1]; it often presents as a solitary lesion amenable to surgical resection for symptom relief and potential cure in isolated cases [2]. Radical ‘en bloc’ sternectomy with negative margins is recommended to achieve local control, followed by reconstruction to maintain thoracic stability and protect mediastinal structures [3].

The procedure of total sternectomy, which involves the complete surgical removal of the sternum, is notably complex. Following this procedure, reconstructing the anterior chest wall is vital to restore structural integrity, protect the intrathoracic organs, and maintain proper respiratory function [4]. Several reconstruction techniques are available, such as wire-only closure, mesh, rigid plates, and combinations of these methods [5]. Each technique offers unique considerations in terms of effectiveness, safety, technical complexity, and long-term outcomes [6].

We describe a unprecedented and cost-effective multidisciplinary approach for managing a case of solitary sternal metastasis from papillary thyroid carcinoma with total sternectomy and chest wall reconstruction using a modified Robicsek stainless steel wire-only technique without prosthetic implantation.

## Case presentation

A 40-year-old man was referred to our unit at the Bangladesh Medical University with a painful and enlarging mass on the anterior chest and a concurrent thyroid nodule. Positron emission tomography-computed tomography (PET-CT) identified a solitary hypermetabolic lytic lesion affecting the entire sternum and a fluorodeoxyglucose (FDG)-avid lesion in the right thyroid lobe (Figure 1).

**Figure 1:**
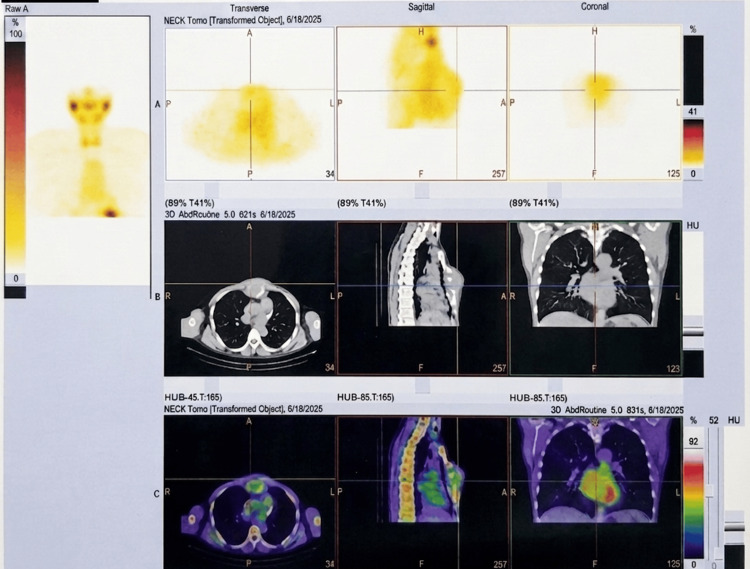
Thyroid scan with single photon emission computed tomography-computed tomography (SPECT-CT) of neck and chest A) Thyroid scan of the neck showed a mildly enlarged thyroid gland with homogeneous radiotracer distribution. The central chest focal area of the radiotracer concentration was observed. B + C) Chest CT shows a large lytic lesion in the manubrium sternum with adjoining large soft tissue mass formation measuring about 6.0x5.8 cm

A preoperative biopsy confirmed the presence of metastatic papillary thyroid carcinoma (Figure 2). 

**Figure 2:**
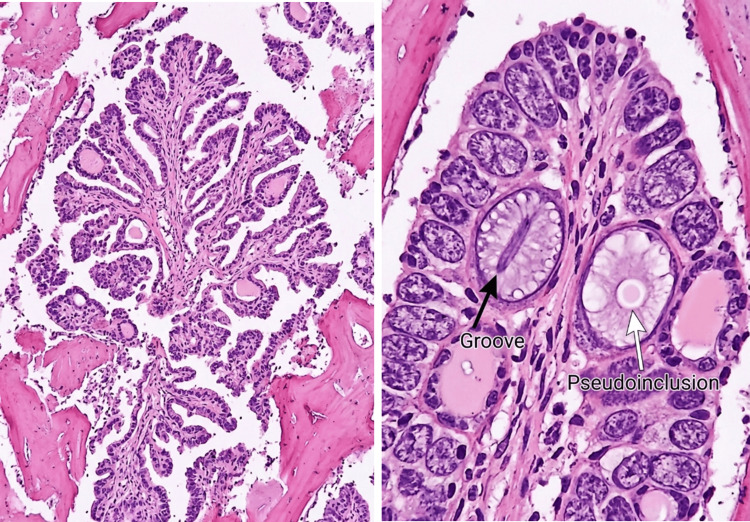
Tissue from manubrium sterni Shows papillary thyroid carcinoma invasion of adjacent bony tissue. In the higher magnification view, focusing on the details of the cell nuclei, it shows Nuclear Grooves marked with the black arrow and Intranuclear Pseudoinclusions marked with white arrows, where the pink cytoplasm indents the nucleus

Following a multidisciplinary consultation involving an oncologist, anaesthetist, ENT specialist, and cardiothoracic surgeon, the patient underwent six cycles of paclitaxel-carboplatin chemotherapy to optimise systemic control prior to surgery. Subsequent restaging imaging revealed no additional metastatic disease.

After preoperative optimisation, the patient was evaluated by an anaesthesiologist, and on October 10, 2025, the patient underwent endotracheal general anaesthesia with standard American Society of Anesthesiologists (ASA) monitoring. An ENT surgeon performed a total thyroidectomy, ensuring the preservation of the recurrent laryngeal nerves and parathyroid glands. Subsequently, the thoracic surgery team conducted a radical 'en bloc' total sternectomy. Meticulous preoperative planning was conducted for the total sternectomy (Figure 3).

**Figure 3:**
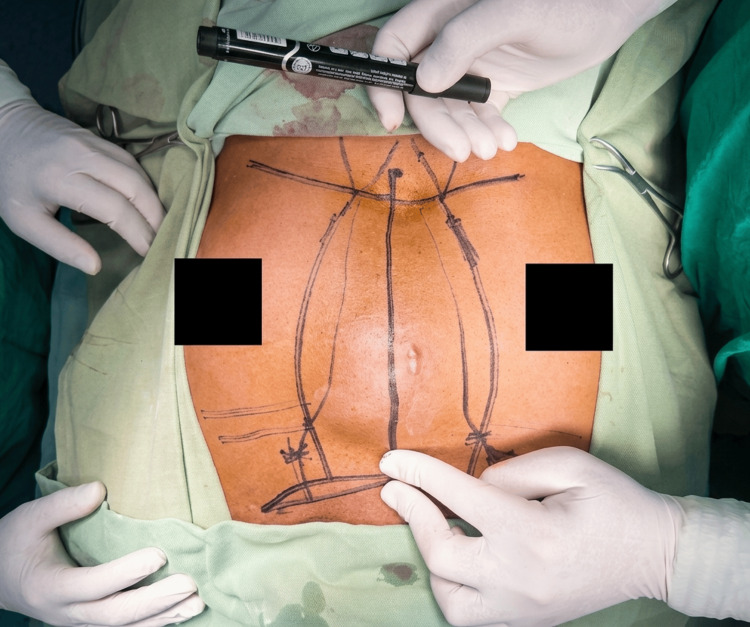
Presurgical planning

The resection encompassed the manubrium, sternal body, xiphoid, and the medial ends of both clavicles, as well as the first to seventh costal cartilages (Figure 4).

**Figure 4:**
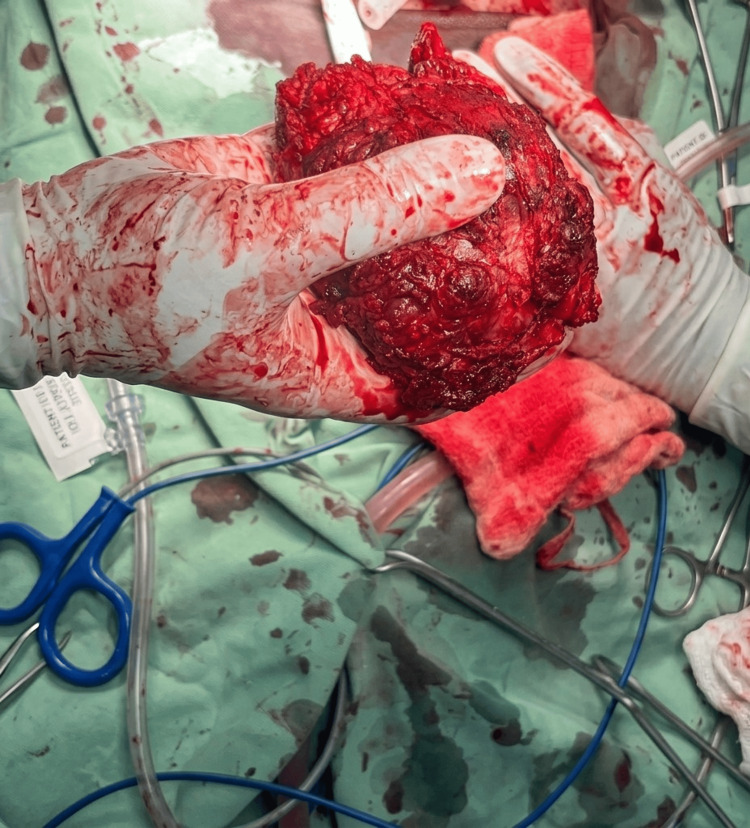
The specimen removed after an en-bloc total sternectomy comprised the manubrium, the sternal body, the xiphoid process, and the medial heads of the clavicles

Confronted with a substantial 15 × 10-cm defect, we implemented a modified Robicsek technique utilising stainless steel No. 5 sternal wires. Longitudinal wires were expertly interwoven through the bilateral costal cartilages to establish stable lateral "pylons." Subsequently, transverse wires were threaded through these weaves across the midline, forming a tension-balanced semi-rigid "neosternum" in accordance with the biomechanical principles of the Robicsek technique (Figure 5).

**Figure 5:**
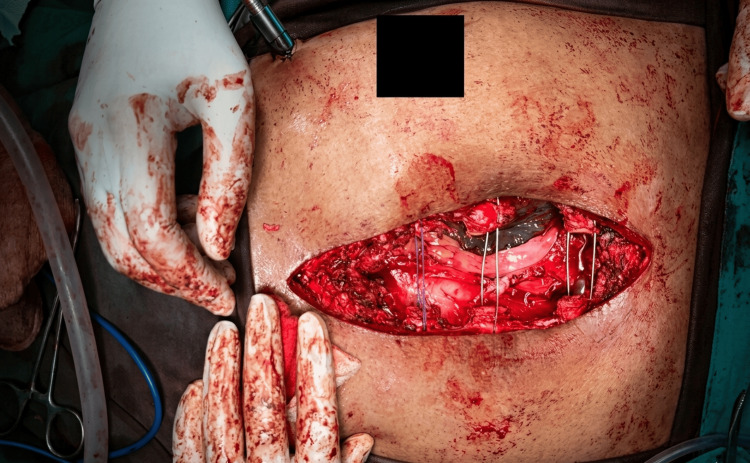
The intraoperative perspective of the modified Robicsek reconstruction shows longitudinal parasternal wires interlaced through the costal cartilages, while transverse wires extend across the midline to form a semi-rigid anterior structure

Notably, we did not use any prosthetic mesh or muscle flaps in this procedure. To address the considerable skin tension following the approximation of the thoracic cage, deep vertical mattress (Donati) stitches were employed for primary closure (Figure 6).

**Figure 6:**
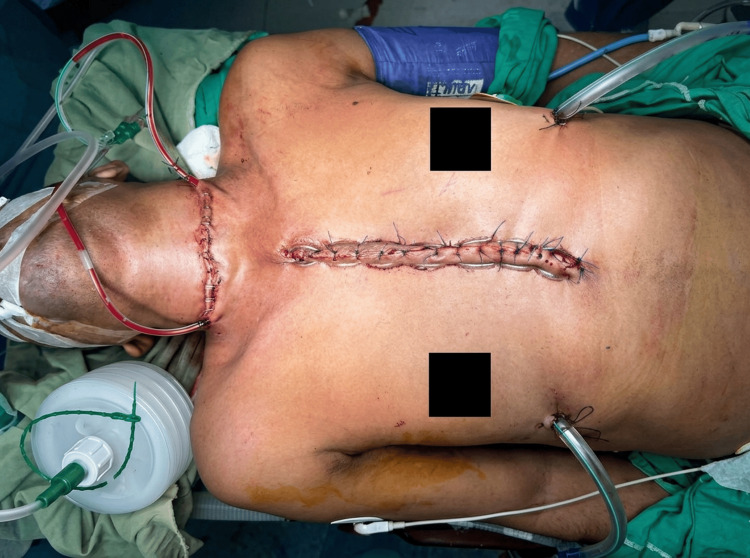
Primary closure of the soft tissue was accomplished by employing deep vertical mattress sutures, known as the Donati technique, to minimize tension along the incision on the anterior chest wall

The patient was extubated six hours postoperatively. Postoperative clinical evaluation indicated no evidence of paradoxical respiration or chest wall instability. Skin stitches were removed on the 15th day, demonstrating healing by primary intention. A follow-up at one month confirmed complete functional recovery.

## Discussion

Total sternectomy for solitary sternal metastases requires reconstruction to restore thoracic stability, protect mediastinal structures, and preserve respiratory function.

Rigid plate fixation (RPF) provides enhanced mechanical stability and is associated with reduced sternal wound complications and reoperations in high-risk patients [7]. However, RPF is also associated with an increased bleeding rate (28.9%), prolonged ventilation (16.5%), and reoperation/readmission (15.2%) [8].

Mesh-based reconstructions, including biosynthetic absorbable mesh, demonstrate efficacy in contaminated surgical fields, with a postoperative surgical site infection rate of 45.7% and a hernia recurrence rate of 10% at a median follow-up of 20 months [9].

Musculocutaneous and omental flap reconstructions following sternectomy due to infection are utilized for complex defects [10]; however, existing literature reports no perioperative mortality but high morbidity affecting 57% of patients in one series, and omental flaps may have complication rates like hernias up to 32% or postoperative bleeding up to 15% [11]. Combined techniques, such as plates with mesh or muscle flaps, are typically reserved for addressing extensive defects or deep infections, offering the potential for definitive infection control.

Wire-only closure is associated with variable rates of sternal stability, with outcomes influenced by factors such as sternal quality, patient risk factors, and chosen closure techniques [12]. Single-wire techniques have demonstrated higher rates of sternal instability (14.7%) compared to double-wire (4%) or figure-of-8 configurations (1.3% vs. 0.1%) [13]. This instability can potentially lead to postoperative complications, including lung dysfunction, sternal non-union or dehiscence, and chest wall fat liquefaction [14]. In humans, titanium wires are non-inferior to stainless steel wires regarding sternal instability (3.7% vs. 5.0%) and wound infection, with lower postoperative pain in the titanium group [15]. Wire-only closure, as a traditional sternal closure method, is generally straightforward and cost-effective; however, achieving proper tension and placement is critical to prevent instability, particularly in high-risk patients [16].

## Conclusions

Our modified Robicsek stainless steel wire-only method ensures balanced deep-layer opposition and superior edge eversion, both critical for preventing dehiscence in high-tension thoracic wounds. This unprecedented procedure showed excellent postoperative results, with no paradoxical respiration, chest wall instability, or other complications during clinical assessment.

## References

[1] Şenol Z, Gülşen T (2021). Papillary thyroid carcinoma with rare metastasis to Costa and vertebrae: case report. J Cukurova Anesth Surg.

[2] Riker AI, Hodgdon IA, Dewenter TA, Marshall R, Boulmay B (2021). Metastatic papillary thyroid cancer to the liver: the central role of a multidisciplinary approach to treatment. Ochsner J.

[3] Aramini B, Masciale V, Radaelli LF, Sgarzani R, Dominici M, Stella F (2022). The sternum reconstruction: present and future perspectives. Front Oncol.

[4] Aranda JL, Gomez MT, Fuentes M, Rivas C, Forcada C, Jimenez MF (2024). Sternal resection and reconstruction: a review. J Thorac Dis.

[5] Baccarani A, Filosso P, Marra C (2023). Reconstruction of complex anterior chest wall defects: the lasagna technique. Plast Reconstr Surg Glob Open.

[6] Colella S, Brandimarte A, Marra R (2022). Chest wall reconstruction in benign and malignant tumors with non-rigid materials: An overview. Front Surg.

[7] Park JS, Kuo JH, Young JN, Wong MS (2017). Rigid sternal fixation versus modified wire technique for Poststernotomy closures: a retrospective cost analysis. Ann Plast Surg.

[8] Tran BN, Chen AD, Granoff MD (2019). Surgical outcomes of sternal rigid plate fixation from 2005 to 2016 using the American College of Surgeons-National Surgical Quality Improvement Program database. Arch Plast Surg.

[9] Claessen JJ, Timmer AS, Atema JJ, Boermeester MA (2021). Outcomes of mid-term and long-term degradable biosynthetic meshes in single-stage open complex abdominal wall reconstruction. Hernia.

[10] Jo GY, Ki SH (2023). Analysis of the chest wall reconstruction methods after malignant tumor resection. Arch Plast Surg.

[11] Kim RY, Lee CJ, Yoo G (2025). Reconstruction of anterior chest wall defect using an omental flap and split-thickness skin graft: a case report. J Wound Manag Res.

[12] Shrestha O, Bohara S, Chettri ST (2025). Outcomes of rigid fixation compared to wire closure after median sternotomy a systematic review and meta-analysis. Ann Med Surg (Lond).

[13] Shafi AM, Abuelgasim E, Abuelgasim B, Iddawela S, Harky A (2021). Sternal closure with single compared with double or figure of 8 wires in obese patients following cardiac surgery: a systematic review and meta-analysis. J Card Surg.

[14] Yu Y, Xia J, Jiang Q (2024). Causes of mediastinitis and its surgical treatment. Heart Surg Forum.

[15] Çelik E, Çora AR (2023). Does elective sternal plating combined with steel wire reduce sternal complication rates in patients with obesity?. Braz J Cardiovasc Surg.

[16] Liu J, Chen X, Ma G, Liu X, Li X, Miao Q, Zhang C (2022). Comparison of sternal fixation strategies after open-heart surgery via a median sternal incision. Heart Surg Forum.

